# Association of Health-Related Quality of Life with Functional and Radiological Outcomes in Patients with Rheumatoid Arthritis–Associated Interstitial Lung Disease, Systemic Sclerosis–Associated Interstitial Lung Disease, and Idiopathic Pulmonary Fibrosis

**DOI:** 10.5152/ArchRheumatol.2025.11105

**Published:** 2025-09-01

**Authors:** Irem Sahinoğlu, Deniz Kizilirmak, Sadettin Uslu, Mevlüt Kacar, Filiz Cemre Tasgöz, Seref Sülükcü, Emre Ali Acar, Müge Gencer Tuluy, Secil Sari, Ozgül Soysal Gündüz, Timur Pirildar

**Affiliations:** 1 Department of Rheumatology, Celal Bayar University School of Medicine, Manisa, Türkiye; 2 Department of Chest Diseases, Celal Bayar University School of Medicine, Manisa, Türkiye; 3 Department of Physiotherapy, Celal Bayar University School of Medicine, Manisa, Türkiye

**Keywords:** Idiopathic pulmonary fibrosis, interstitial lung disease, rheumatoid arthritis, Saint George Respiratory Questionnaire, systemic sclerosis, Warrick scoring system

## Abstract

**Background/Aims:**

This study aimed to investigate the association between lung function and imaging parameters with health-related quality of life (HRQoL), as measured by the St. George’s Respiratory Questionnaire (SGRQ), in patients with rheumatoid arthritis–associated interstitial lung disease (RA-ILD), systemic sclerosis–associated ILD (SSc-ILD), and idiopathic pulmonary fibrosis (IPF).

**Materials and Methods:**

In this cross-sectional study, a total of 120 patients (37 RA-ILD, 42 SSc-ILD, and 41 IPF) were included. Demographic, clinical, functional (forced vital capacity [FVC], lung diffusion capacity for carbon monoxide [DLCO], 6‑minute walk test), and radiological (Warrick scores) data were collected. The associations between SGRQ scores and these parameters, as well as other patient-reported outcome measures (PROMs), were analyzed.

**Results:**

St. George’s Respiratory Questionnaire scores showed significant correlations with functional measures and PROMs across all groups. However, no correlation was found between SGRQ and FVC only in RA-ILD. In SSc-ILD and IPF, SGRQ scores were also significantly associated with high-resolution computed tomography–based Warrick scores. However, no correlation was found between SGRQ and radiological parameters in RA-ILD. Receiver operating characteristic (ROC) analysis demonstrated that SGRQ could help identify patients with impaired lung function (FVC <70%) in IPF and SSc-ILD groups.

**Conclusion:**

St. George’s Respiratory Questionnaire may be a valuable tool for evaluating HRQoL in patients with SSc-ILD and IPF, with moderate associations with functional and radiological outcomes. Its utility in RA-ILD appears to be more limited and requires further investigation.

**MAIN POINTS**

- In SSc-ILD and IPF, SGRQ scores showed moderate to strong correlations with FVC, DLCO, 6MWT, and Warrick scores.
- In RA-ILD, SGRQ scores did not correlate with FVC or HRCT scores, and only the SGRQ symptom score and DLCO had a negative correlation.
- IPF patients had significantly worse SGRQ, HAQ, and SF-12 scores compared to RA-ILD and SSc-ILD patients.
- SGRQ correlated strongly with SF-12 and HAQ in all groups, supporting its use as a PROM in ILD.Type the Paragraph Text

## Introduction

Interstitial lung disease (ILD) is a common lung manifestation of connective tissue disease (CTD) that is associated with both morbidity and mortality.^1^ Interstitial lung disease is 70%-90% common in patients with systemic sclerosis (SSc), and up to 45% of cases are complicated by moderate-to-severe ILD. Due to a lack of screening, the incidence of ILD in CTDs other than SSc is unknown.^2^ However, it is known that approximately 10% of patients with rheumatoid arthritis (RA) have ILD, and it causes significant morbidity and mortality.^3^

In addition to CTD-related ILDs, idiopathic pulmonary fibrosis (IPF) is another specific type of ILD that causes significant morbidity and mortality. IPF, a fatal lung disease, is characterized by a chronic progressive interstitial pneumonia etiology and is associated with the characteristic histological and/or radiological pattern of interstitial pneumonia.^4^

Connective tissue disease patients tend to be younger and more likely to be female than IPF patients. Patients with CTD-ILD have a 5-year survival rate of almost 70%, compared to 20%-35% for patients with IPF, which is a worse prognosis than CTD-ILD.^5,6^ Measures of disease severity at diagnosis, such as lung function decline and degree of fibrosis on high-resolution computed tomography (HRCT), are most strongly associated with mortality.^7^ Physicians commonly use a variety of conventional outcome measures, including HRCT scans, pulmonary function tests (PFTs), and the 6-minute walk test (6-MWT), to monitor the clinical course of ILD patients and evaluate the effectiveness of their therapy.^8^

Currently, there is no consensus on measures to be used to assess disease activity or treatment response in CTD-ILD and IPF.^9^ Impaired lung function and related symptoms make it difficult to carry out everyday tasks and have a negative impact on health-related quality of life (HRQoL). According to qualitative research, patients perceive the objective measures used in ILD clinics as disconnected from their actual experience of the disease.^10,11^ The use of patient-reported outcome measures (PROMs) to measure HRQoL decline in lung disease is becoming more widely accepted. The incorporation of patient narratives, which have been generally ignored in modern medicine, may be made possible by the use of PROMs as endpoints in research studies and as tools for therapeutic follow-up. Patient-reported outcome measures should be viewed as a complement to established measures of effectiveness, not a substitute for them.^12^ The St George’s Respiratory Questionnaire (SGRQ) is the most popular of the other PROMs available. It has been used extensively to assess HRQoL in patients with IPF and CTD-ILD, although it was originally developed for use in patients with chronic obstructive pulmonary disease.^13,14^

There are few studies evaluating the relationship between functional, radiological, and PROMs in RA-ILD, SSc-ILD, and IPF patients in real-world datasets, and there is no previous study comparing these 3 specific groups. This study’s objective was to look into the correlation between PROMs in patients with RA-ILD, SSc-ILD, and IPF, including both functional and radiological characteristics.

## Methods

### Study Population

The study included patients (18 years of age and older) who fulfilled the ACR/EULAR classification criteria^15,16^ for the diagnosis of SSc and RA and the 2011 American Thoracic Society guidelines for the diagnosis of IPF.^4^ Demographic characteristics and clinical and laboratory findings were recorded. The modified Rodnan skin score (mRSS)^17^ was used to measure the degree of skin involvement in SSc while the disease activity score 28 (DAS 28)^18^ was used to measure the disease activity in RA. Patients were asked to complete patient-based questionnaires; SGRQ, 12-item Short Form Health Survey (SF-12), and Health Assessment Questionnaire (HAQ) at enrolment. All patients were enrolled during routine clinic visits. Patients who did not have PFTs, HRCT, and a 6MWT within 6 months of completing the patient-based questionnaires were excluded from the study. Individuals with overlap syndromes, chronic lung diseases other than IPF, SSc-ILD, or RA-ILD, cognitive impairment, active pulmonary infection, pulmonary arterial hypertension, uncontrolled congestive heart failure, and other conditions were also excluded.

#### St. George’s Respiratory Questionnaire:

The symptom scale assesses the severity of respiratory symptoms. The activity scale assesses the impact of respiratory problems on a person’s ability to function. The Impact Scale assesses how respiratory problems affect daily activities and general health. The scores for each of the 3 domains range from 0 to 100. All 3 parts are used to calculate the total scale. Higher scores indicate poorer HRQoL.^13^

#### Health-Related Quality of Life Short Form-12:

The 8 dimensions of health status assessed by 12 questions are general health, physical functioning (PF), physical role (RP), bodily pain, vitality (VT), social functioning (SF), emotional role (RE), and mental health (MH). While the rest have only one of these items, SF, RP, RE, and MH have 2. Each item has between 2 and 6 levels. The dimension data can also be used to generate 2 summary scores: the Mental Component Summary and the Physical Component Summary. Higher scores indicate better HRQoL. Both scores range from 0 to 100.^19^

#### Health Assessment Questionnaire:

The Health Assessment Questionnaire (HAQ) assesses a patient’s functional ability using 20 questions divided into 8 categories: dressing, getting up, eating, walking, hygiene, reaching, grasping, and activities of daily living. Each category has 2 or 3 items, with a 4-point rating system applied to each item (0 indicates no difficulty, 1 indicates significant difficulty, 2 indicates severe disability, and 3 indicates incapacity). The total HAQ score is the average of the 8 categories on a scale of 0 to 3. The score for each category is the highest score for the item in that category.^20^

#### High-Resolution Computed Tomography of the Lung:

High-resolution computed tomography (HRCT) scans were reviewed by an experienced pulmonologist (K.D.) who was blinded to clinical and other trial data. The Warrick scoring system was used to determine the severity score, extent score, and total score of parenchymal involvement for each patient. This score is 1 point for ground glass opacity, 2 points for irregular pleural margins, 3 points for septal or subpleural lines, 4 points for honeycombing, and 5 points for subpleural cysts. The severity score of parenchymal involvement is obtained by adding the scores of the findings on the HRCT images. The maximum severity score is 15. To calculate the extent score of parenchymal involvement, the number of lung segments in which each lesion is located is determined. If the specified lesions are present in 1-3 segments, 1 point is given; if they are present in 4-9 segments, 2 points are given; if they are present in more than 9 segments, 3 points are given, and the extent score of parenchymal involvement is obtained by adding the points given. The maximum score for the extent score is 15. The total Warrick score is obtained by adding the extent score and the severity score. The maximum score for the total Warrick score is 30.^21^

#### Pulmonary Function Testing and 6‑Minute Walk Test:

Forced vital capacity (FVC) and lung diffusion capacity for carbon monoxide (DLCO) were assessed by spirometry. The 6MWT was used to determine functional submaximal exercise capacity. Patients were instructed to walk for 6 minutes and the distance covered was recorded.^22^

### Compliance with Ethical Standards

The study was approved by the Ethics Committee of Manisa Celel Bayar University (Date: November 8, 2023; Decision No.: 2068), and all participants provided signed informed permission. In all cases involving human subjects, the Helsinki Declaration of 1964 and its updates, comparable ethical standards, or the ethical mandates of the national and/or institutional research committee have been followed. Informed consent was obtained from all participants prior to their inclusion in the study. 

### Statistical Analysis

SPSS version 20.0 software (IBM SPSS Corp.; Armonk, NY, USA) was provided by IBM Inc, Chicago, IL, USA, for statistical analysis. Normality was assessed using the Shapiro–Wilk and Kolmogorov–Smirnov tests. While categorical data are expressed as n (%), quantitative variables are expressed as mean ± SD and median (min./max.). When comparing 3 or more independent groups, ANOVA, and Kruskal–Wallis tests were used for normally distributed and non-normally distributed variables, respectively. The degree of relationship between the radiographic (the amount of pulmonary fibrosis on chest computed tomography scan), functional (FVC, DLCO, 6-MWT), and PROMs (HAQ, SF-12, and SGRQ) were examined using Spearman’s rank correlation coefficients. Less than or equal to 0.29, 0.30 to 0.49 and higher than or equal to 0.50 were regarded as low, moderate, and high correlations, respectively.^23^ The ROC curve was used to assess how well the SGRQ identified individuals with FVC less than 70%. Statistical significance was set at *P* < .05.

## Results

### Demographics

Forty-two patients with SSc-ILD, 92.9% (n = 39) of whom were female with a median age of 56 years (range: 27-77), 37 patients with RA-ILD, 81.1% (n = 30) of whom were female with a median age of 64 years (range: 42-82) and 41 patients with IPF, 26.8% (n = 11) of whom were female with a median age of 68 years (range: 50-82) were included (Table 1). Age variable, gender, and disease duration show statistically significant differences (*P* < .001, *P* < .001, and *P* = .001 respectively) between the 3 groups. In the post-hoc analysis, the difference in age was due to SSc and the difference in disease duration was due to IPF. With regard to smoking and the presence of co-morbidities, there is no discernible difference between the 2 groups.

### High-Resolution Computed Tomography Findings, Pulmonary Function Tests–6-Minute Walk Test Results, and Patient-Reported Outcome Measures of Patients

Forced vital capacity, DLCO, and 6MWT results were better in the RA-ILD and SSc-ILD groups compared to IPF (Table 2). In the post-hoc analysis, the difference in FVC and DLCO was due to IPF. Six-minute walk test results had significant differences in RA-ILD compared to IPF. High-resolution computed tomography scores (Warrick total/severity scores) were similar in the 3 groups. The IPF group showed higher SGRQ symptom, impact, activity, total score, and HAQ scores compared to the RA-ILD and SSc-ILD groups. However, there was no statistically significant difference in the SGRQ activity score. In SF-12, both physical and mental scores were better in RA-ILD and SSc-ILD groups compared to IPF.

### Patient-Reported Outcome Measures and Other Variables Correlations in Patients with Rheumatoid Arthritis–Associated Interstitial Lung Disease

Total SGRQ score was associated with 6-MWT, HAQ, DAS 28-ESR, SF-12 physical and mental score (r = −0.565, *P* < .001, r = 0.646, *P* < .001, r = 0.471, *P* = .003, r = −0.773, *P* < .001 and r = −0.607, *P* < .001, respectively). Except for the SGRQ impact score (correlated only with HAQ and SF-12 physical and mental scores), symptom and activity scores showed similar correlations (Table 3). None of the SGRQ scores showed any correlation with Warrick scores and FVC. Only the SGRQ symptom score and DLCO had a negative correlation (r = −0.470, *P* = .003).

### Patient-Reported Outcome Measures and Other Variables Correlations in Patients with Systemic Sclerosis–AssociatedInterstitial Lung Disease

Total SGRQ score was associated with FVC, DLCO, 6-MWT, HAQ, SF-12 physical and SF-12 mental scores (r = −0.456, *P *= .002, r = −0.620, *P* < .001, r = −0.488, *P* = .001, r = 0.599, *P* < .001, r = −0.726, *P* < .001 and r = −0.650, *P* < .001, respectively). Warrick severity/extent/total scores were correlated with the total SGRQ score (r = 0.511, *P* = .001, r = 0.575, *P* < .001, and r = 0.558, *P* = .001, respectively). St. George’s Respiratory Questionnaire symptom, activity, and impact scores showed similar correlations (Table 3).

### Patient-Reported Outcome Measures and Other Variables Correlations in Patients with Idiopathic Pulmonary Fibrosis

Total SGRQ score was associated with FVC, DLCO, 6-MWT, HAQ, SF-12 physical and SF-12 mental scores (r = −0.618, *P* < .001, r = −0.535, *P* < .001, r = −0.607, *P* < .001, r = 0.612, *P* < .001, r = −0.467, *P* = .002 and r = −0.521, *P* < .001 respectively). Total SGRQ score was associated with Warrick severity/extent/total scores (r = 0.584, *P* < .001, r = 0.552, *P* < .001, and r = 0.586, *P* = <.001, respectively). St. George’s Respiratory Questionnaire symptom, activity, and impact scores showed similar correlations (Table 3).

### Results of the ROC Curve Study Used to Assess How Well the St. George’s Respiratory Questionnaire Performed in Idiopathic Pulmonary Fibrosis Patients

**Figure 1.**
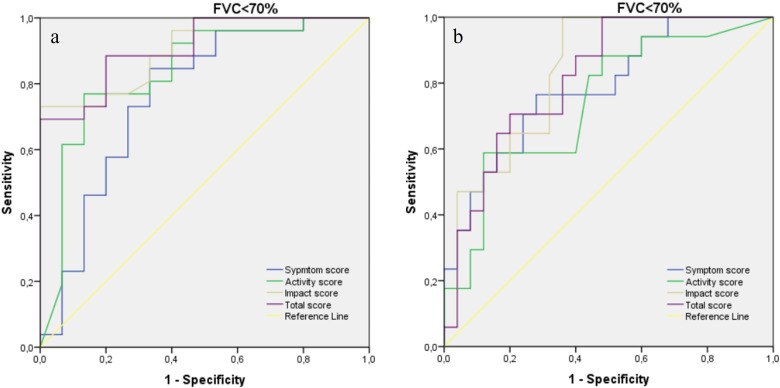
The results of ROC curve analysis for evaluating the performance of St. George’s Respiratory Questionnaire in patients with (a) idiopathic pulmonary fibrosis and (b) systemic sclerosis–associated interstitial lung disease.

The whole SGRQ, symptom, activity, and impact scores had fair results [AUC 95% (confidence interval, CI): 0.910 (0.825-0.996), 0.764 (0.598-0.930), 0.837 (0.700-0.975), 0.906 (0.819-0.994) and *P* values <.001, .005, <.001, <.001 respectively] according to the findings of the ROC curve analysis used to determine the existence of patients with FVC below 70% (Figure 1a). The cutoff score was determined by using the SGRQ total score for FVC < 70%. A total score of 63.26 showed a sensitivity of 80% and specificity of 80%.

### Results of the ROC Curve Study Used to Assess How Well the St. George’s Respiratory Questionnaire Performed in Systemic Sclerosis–Associated Interstitial Lung Disease Patients

The whole SGRQ, symptom, activity, and impact scores had fair results [AUC 95% (confidence interval, CI): 0.816 (0.690-0.943), 0.786 (0.646-0.925), 0.740 (0.585-0.895), 0.834 (0.713-0.955) and *P* values .001, .002, .009, <.001 respectively] according to the findings of the ROC curve analysis used to determine the existence of patients with FVC below 70% (Figure 1b). The cutoff score was determined by using the SGRQ impact score for FVC < 70%. A total score of 41.20 showed a sensitivity of 70% and specificity of 68%.

## Discussion

The results of this study showed different levels of association between the SGRQ and different types of outcomes. Specifically, a relationship was found between FVC and the total SGRQ as well as the sub-items of the SGRQ that measure symptoms, activities, and impact scores in the SSc-ILD and IPF patient groups. Nevertheless, no such correlation was discovered between FVC and the total SGRQ as well as its sub-items in RA-ILD patients. Furthermore, this study showed that RA-ILD and SSc-ILD patients had better HRQoL measured by SGRQ, HAQ, and SF-12 compared with patients with IPF. This may suggest that younger age and better lung function may contribute to better HRQoL in SSc-ILD and RA-ILD patients compared to IPF patients.

Forced vital capacity is a prominently recognized and extensively utilized outcome metric in both clinical practice and Randomized Controlled Trials for the diagnosis and follow-up of patients with ILD.^24^ Forced vital capacity has been shown to correlate with the total SGRQ and the activity score sub-item of the SGRQ, but not with symptom or impact ratings, according to a recent study conducted in patients with SSc-ILD. In addition, no correlation was found between 6MWT and any of the SGRQ scores in this study.^8^ This result may be due to the inclusion of patients with pulmonary hypertension who may have confounded the 6MWT results. In this study, individuals with pulmonary hypertension were deliberately excluded from the analysis to create as homogeneous a group of patients as possible without concomitant confounding variables that may have further influenced the 6MWT, regardless of the degree of lung fibrosis. Conversely, among individuals with SSc-ILD, this research demonstrated a correlation between FVC, DLCO, and 6MWT, and the total and subscale SGRQ scores.

The SGRQ has demonstrated construct validity in patients with SSc-ILD. The SGRQ was assessed in 28 individuals with SSc-ILD by Beretta et al.^25^ Correlations were found between 6MWT and SGRQ activity, effect, and total scores (r = −0.590, *P* < .001, r = −0.860, *P* < .005, r = −0.770, *P* < .001, respectively) and between FVC and activity score (−0.470, *P* < .05). Additionally, this study demonstrated a relationship between activity, impact, and total scores with the severity of the disease on HRCT. Thirty-three SSc-ILD patients were assessed by Sözener et al.^26^ who found a negative association (r = −0.496, *P* = .005) between SGRQ activity score and DLCO. They also found a negative correlation between the 4 scales of the SGRQ and 6MWT (r = −0.685, *P* < .001). In the current study, all domains of the SGRQ were correlated with both DLCO and FVC. In a study by Wallace et al.^27^ with a larger number of SSc patients, it was reported that the total SGRQ score was associated with FVC, DLCO, 6MWT, and HAQ (r = −0.380, *P* < .001, r = −0.550, *P* < .001, r = −0.290, *P* = .040, and r = 0.380, *P* < .001, respectively). St. George’s Respiratory Questionnaire symptom, activity, and impact scores showed similar correlations. These results were similar to the current study.

Forced vital capacity is frequently the main outcome measure in SSc-ILD clinical studies; however, from the patient’s point of view, changes in FVC may not always translate into clinically significant benefits. For instance, there was no correlation between baseline SGRQ and baseline FVC in the Scleroderma Lung Study II findings. On the other hand, there was a weak correlation between increases in SGRQ scores and increases in FVC. The quantitative measures of fibrosis, ILD, and DLCO showed a strong baseline correlation with SGRQ. These results, along with the current study, suggest that these additional assessment tools may provide a more complete picture of the patient’s experience of the disease and may help guide treatment decisions in determining the severity of SSc-ILD.^28^

Previous cross-sectional studies in patients with IPF have examined the relationship between the 6MWT and the SGRQ total and domain scores. Correlations were moderate to high for the total, impact, and activity scores, but modest for the symptom score.^29-31^ Similar correlations were observed in the current study. The symptoms domain had a weaker correlation than the other domains. This may be because cough and breathlessness are the main symptoms of IPF patients, although a wide range of respiratory symptoms (cough, sputum, shortness of breath, wheezing, and episodes of chest pain) were asked. St. George’s Respiratory Questionnaire total/domain scores and FVC have been shown to have weak to moderate associations in the same studies. In the current study, the correlation between the SGRQ total/activity/impact score and FVC/DLCO was moderate to strong. Total HRCT scores and each SGRQ component showed a significant association (r > 0.3, *P* < .001) according to Peng et al.^29^ In the current study, the correlations between HRCT total scores and each SGRQ component were also moderate to high.

In a previous study in which RA-ILD was specifically considered in the context of CTD, only FVC was significantly correlated with the SF-36 role function/physical score (r = 0.724, *P* < .001) in RA-ILD patients.^32^ In this study, there was no correlation between PFTs and SGRQ. The researchers found no significant correlation between SGRQ and PFTs and HRCT measurements in RA-ILD patients. These findings were similar to the current study. They found significant correlations between PROMs in their study. In the current study, the SGRQ and other PROMs showed a good correlation. They did not examine the SGRQ with all domains, but only the total SGRQ score and its relationship with other measures. For these reasons, the current study is the first to evaluate the SGRQ with all domains separately in RA patients. This study did not analyze the relationship between DAS 28-ESR scores and the SGRQ. In this study, we evaluated RA patients were evaluated in detail, and a relationship was found between the DAS 28-ESR score and the total symptom/activity domains of the SGRQ. This finding was not surprising, as joint symptoms and high disease activity negatively affect the quality of life in RA patients.^33^ Another finding that was not investigated in this study was exercise capacity as measured by the 6MWT. In the current study, a correlation was found between SGRQ and 6MWT, whereas no significant correlation was found between SGRQ and HRCT scores and pulmonary function tests. This may be because the exercise capacity test may differ not only depending on the lung involvement but also depending on the joint pain and stiffness of the patients. It was also shown that RA-ILD and SSc-ILD patients had better HRQoL, as measured by both physical and mental scores with the SGRQ, HAQ, and SF-12, compared to patients with IPF. Rheumatoid arthritis and SSc patients were younger, female, and had better lung function as measured by FVC, DLCO, and 6MWT than IPF patients. St. George’s Respiratory Questionnaire symptom, impact, activity, and total scores were worse in IPF. However, the activity domain was not statistically different between IPF and RA-ILD and SSc-ILD. Although lung function parameters were worse in IPF, the SGRQ activity score did not differ significantly. This may be explained by the contribution of musculoskeletal involvement to the activity score in both RA and SSc.

According to the physical component score of the SF-36, RA-ILD patients had lower HRQoL than IPF patients according to a previous study by Natalini et al.^34^ In this study, HRQoL differences between these 2 subtypes of ILD are partly explained by the severity of breathlessness and having stiffness or discomfort in joints. Durheim et al.^35^ analyzed HRQoL between IPF and SSc-ILD patients. They found that HRQoL was similar between IPF and SSc-ILD patients after adjustment for age, sex, and lung function. However, unadjusted ILD-specific HRQoL was better in SSc-ILD than in IPF. In the current study, SSc-ILD patients and RA-ILD patients were younger and had better lung function. This may suggest that younger age and better lung function may contribute to better HRQoL in SSc-ILD and RA-ILD patients compared to IPF.

This study has several limitations. As the study was cross-sectional, changes in the SGRQ at follow-up were not recorded. This study did not assess the responsiveness of SGRQ over a longer period of time. One such limitation is the small number of patients. Despite these limitations, this study may still be helpful to other published studies because it included 3 different groups of patients. The SGRQ as a component of HRQoL in RA-ILD patients has not been thoroughly investigated until this study.

In conclusion, these results confirm the value of the SGRQ as a PROM and provide further insight into lung involvement in SSc-ILD and IPF, but not in RA. Further studies are needed to determine whether the SGRQ can assess changes over time or in response to therapy in patients with RA-ILD, SSc-ILD, and IPF.

## Figures and Tables

**Table 1.** Demographics and Clinical Characteristics of All Patients

	RA-ILD (n = 37)	SSc-ILD (n = 42)	IPF (n = 41)	*P*
Age, years	64 (42-82)	56 (27-77)	68 (50-82)	<.001
Female	30 (81.1)	39 (92.9)	11 (26.8)	<.001
Disease duration, months	107.29 ± 111.78	118.40 ± 89.38	45.95 ± 28.76	.001
BMI	27.40 ± 4.52	25.97 ± 5.55	24.93 ± 4.08	.078
Smoking	10 (27.0)	13 (31.0)	22 (53.7)	.065
HT	14 (37.8)	18 (42.9)	26 (63.4)	.053
DM	10 (27)	3 (7.1)	8 (19.5)	.062
CAD	4 (10.8)	1 (2.4)	3 (7.3)	.311
HPL	8 (21.6)	3(7.1)	3 (7.3)	.106
Rf positive	22 (59.5)	–	–	–
Anti-CCP positive	19 (51.4)	–	–	–
ACA positive	–	2 (4.8)	–	–
Anti-Scl-70 positive	–	32 (76.2)	–	–
DAS-28	3.26 ± 1.47	–	–	–
mRSS	–	19.19 ± 10.92	–	–

While categorical data are expressed as n (%), quantitative variables are expressed as mean ± SD and median (min./max.).

ACA, anti-centromere antibody; BMI, body mass index; CAD, coronary artery disease; CCP, cyclic citrullinated peptide; DAS-28, Disease Activity Score-28; DM, diabetes mellitus; HL, hyperlipidemia; HT, hypertension; mRSS, modified Rodnan skin score; Rf, rheumatoid factor; Scl-70, anti-topoisomerase-1 antibodies.

**Table 2.** High-Resolution Computed Tomography Findings, Pulmonary Function Tests-6-Minute Walk Test Results, and Patient-Reported Outcome Measures of Patients

	RA-ILD (n = 37)	SSc-ILD (n = 42)	IPF (n = 41)	*P*
Warrick severity score	6.54 ± 4.46	6.04 ± 4.20	7.39 ± 4.12	.299
Warrick extent score	6.08 ± 3.13	5.52 ± 3.07	7.17 ± 2.68	.046
Warrick total score	12.62 ± 7.40	11.57 ± 7.00	14.56 ± 6.63	.110
SGRQ symptom score	43.57 ± 28.27	40.62 ± 29.21	64.32 ± 18.57	<.001
SGRQ activity score	57.21 ± 31.12	53.36 ± 31.19	69.32 ± 22.19	.071
SGRQ impact score	44.00 ± 38.65	36.72 ± 27.05	65.46 ± 19.64	<.001
SGRQ total score	45.21 ± 27.95	42.41 ± 27.02	66.47 ± 18.18	<.001
SF-12 PF	37.24 ± 10.69	35.62 ± 9.51	29.04 ± 8.42	<.001
SF-12 MH	47.24 ± 10.40	40.71 ± 11.03	33.82 ± 9.85	<.001
HAQ	0.74 ± 0.73	0.71 ± 0.66	1.78 ± 0.60	<.001
FVC, % predicted	84.67 ± 17.74	79.07 ± 23.08	63.82 ± 19.10	<.001
DLCO, % predicted	50.44 ± 16.92	48.35 ± 20.33	33.15 ± 15.98	<.001
6MWT	319.45 ± 122.88	286.42 ± 151.38	236.34 ± 134.90	.021

Variables were expressed as mean ± SD.

6-MWT-D, 6-min walk test distance; DLCO, diffusing capacity of the lungs for carbon monoxide; FVC, forced vital capacity; HAQ, health assessment questionnaire; SF-12 MH, 12-item Short Form Health Survey mental health; SF-12 PF, 12-item Short Form Health Survey physical functioning; SGRQ, St George’'s Respiratory Questionnaire.

**Table 3.** The Interrelationship of Other Variables with St. George’s Respiratory Questionnaire

SGRQ								
Total Symptom Activity Impact								
RA-ILD	**r**	***P***	**r**	***P***	**r**	***P***	**r**	***P***
FVC	−0.140	.408	−0.177	.295	−0.158	.351	−0.087	.609
DLCO	−0.258	.123	−0.470	.003	−0.210	.211	0.077	.649
6MWT	−0.565	<.001	−0.403	.013	−0.575	<.001	−0.274	.100
HAQ	0.646	<.001	0.568	<.001	0.578	<.001	0.368	.025
DAS-28	0.471	.003	0.356	.031	0.508	.001	0.263	.116
Warrick severity	0.167	.322	0.174	.304	0.206	.222	0.132	.434
Warrick extent	0.168	.322	0.132	.437	0.185	.272	0.239	.154
Warrick total	0.172	.308	0.161	.342	0.203	.229	0.181	.282
SF-12 PF	−0.773	<.001	−0.571	<.001	−0.721	<.001	−0.390	.017
SF-12 MH	−0.607	<.001	−0.590	<.001	−0.519	.001	−0.320	.054
SSc-ILD								
FVC	−0.456	.002	−0.465	.002	−0.356	.021	−0.466	.002
DLCO	−0.620	<.001	−0.638	<.001	−0.516	<.001	−0.612	<.001
6MWT	−0.488	.001	−0.498	.001	−0.512	.001	−0.413	.007
HAQ	0.599	<.001	0.450	.003	0.655	<.001	0.544	.025
mRSS	0.097	.545	0.042	.795	0.059	.712	0.129	.421
Warrick severity	0.511	.001	0.618	<.001	0.363	.018	0.513	.001
Warrick extent	0.575	<.001	0.590	<.001	0.475	.001	0.570	<.001
Warrick total	0.558	<.001	0.629	<.001	0.426	.005	0.558	<.001
SF-12 PF	−0.726	<.001	−0.606	<.001	−0.721	<.001	−0.687	.017
SF-12 MH	−0.650	<.001	−0.459	.002	−0.625	<.001	−0.544	<.001
IPF								
FVC	−0.618	<.001	−0.366	.019	−0.596	<.001	−0.554	<.001
DLCO	−0.535	<.001	−0.431	.005	−0.521	<.001	−0.519	.001
6MWT	−0.607	<.001	−0.367	.018	−0.527	<.001	−0.607	<.001
HAQ	0.612	<.001	0.587	<.001	0.473	.002	0.607	<.001
Warrick severity	0.584	<.001	0.457	.003	0.555	<.001	0.540	<.001
Warrick extent	0.552	<.001	0.422	.006	0.517	.001	0.512	.001
Warrick total	0.586	<.001	0.455	.003	0.554	<.001	0.543	<.001
SF-12 PF	−0.467	.002	−0.481	.001	−0.389	.012	−0.406	.008
SF-12 MH	−0.521	<.001	−0.463	.002	−0.431	.005	−0.483	.001

6-MWT-D, 6-min walk test distance; DAS-28, Disease Activity Score-28; DLCO, diffusing capacity of the lungs for carbon monoxide; FVC, forced vital capacity; HAQ, health assessment questionnaire; Mrss, modified Rodnan skin score; SF-12 MH, 12-item Short Form Health Survey mental health; SF-12 PF, 12-item Short Form Health Survey physical functioning; SGRQ, St George’s Respiratory Questionnaire.

## Data Availability

The data that support the findings of this study are available on request from the corresponding author.
